# Contrasting metabolic strategies of two co-occurring deep-sea octocorals

**DOI:** 10.1038/s41598-021-90134-5

**Published:** 2021-05-20

**Authors:** M. Rakka, S. R. Maier, D. Van Oevelen, A. Godinho, M. Bilan, C. Orejas, M. Carreiro-Silva

**Affiliations:** 1 IMAR – Instituto do Mar, Universidade dos Açores, Rua Frederico Machado 4, 9901-862 Horta, Portugal; 2 OKEANOS - Instituto de Investigação em Ciências do Mar da Universidade dos Açores, 9901-862 Horta, Portugal; 3 Department of Estuarine and Delta Systems, Royal Netherlands Institute for Sea Research (NIOZ-Yerseke), Yerseke, The Netherlands; 4 Dipartimento di scienze e tecnologie biologiche e ambientali (DiSTeBA), University of Salento, Via Lecce-Monteroni, 73047 Monteroni di Lecce, LE Italy; 5 Centro Oceanográfico de Gijón, Instituto Español de Oceanografia (IEO), Calle de Ramón González Fernández, 70B, 33212 Gijón, Spain

**Keywords:** Animal physiology, Ecophysiology, Marine biology

## Abstract

The feeding biology of deep-sea octocorals remains poorly understood, as attention is more often directed to reef building corals. The present study focused on two common deep-water octocoral species in the Azores Archipelago, *Dentomuricea* aff*. meteor* and *Viminella flagellum*, aiming at determining their ability to exploit different food sources. We adopted an experimental approach, with three different food sources, including live phytoplankton, live zooplankton and dissolved organic matter (DOM), that were artificially enriched with ^13^C and ^15^N (C and N tracers). The presence of tracers was subsequently followed in the coral tissue, C respiration and particulate organic C and N (POC and PON) release. In both species, feeding with zooplankton resulted in significantly higher incorporation of tracers in all measured variables, compared to the other food sources, highlighting the importance of zooplankton for major physiological processes. Our results revealed contrasting metabolic strategies between the two species, with *D.* aff*. meteor* acquiring higher amounts of prey and allocating higher percentage to respiration and release of POC and PON than *V. flagellum*. Such metabolic differences can shape species fitness and distributions and have further ecological implications on the ecosystem function of communities formed by different octocoral species.

## Introduction

Octocorals are common benthic suspension feeders in tropical, subtropical, temperate and polar regions^1–3^. The majority of octocoral species are found in waters deeper than 50 m^4^ where they create dense single- or multi-species aggregations, structuring three-dimensional and highly heterogenous habitats known as coral gardens^3,5^. These communities provide essential habitat for a variety of associated fauna^3,6^.

The Azores Archipelago, located in the central North Atlantic, harbors an extremely rich biodiversity of cold-water octocorals, reaching a total of 101 species which represent the highest octocoral species richness known so far in North Atlantic^7,8^. Coral gardens constitute the most prominent cold-water coral (CWC) habitat in the Azores, with monospecific or multispecific octocoral communities frequently colonizing seamounts and island slopes^7,9^. Because of their life-history traits, including slow growth and high longevity, recovery of octocoral communities from fisheries and other disturbances can be very slow^10,11^ and thus coral gardens have been classified as vulnerable marine ecosystems (VMEs) in need of protection^12,13^. However, effective conservation of VMEs requires knowledge on the species biology and ecology which in the case of deep-sea octocorals is scarce^1^.

Resource acquisition is a key factor in the biology of suspension feeders^14,15^, ultimately determining population dynamics and species distributions^16–18^. Thus, knowledge on feeding biology of key habitat formers such as octocorals is pivotal to understand local ecosystems. Octocorals can feed on a variety of prey including microplankton, nanoeukaryots, as well as detritus^19–22^. In some cases, their diet varies seasonally following the cycles of local phytoplankton and zooplankton communities^15,23^. Although most octocoral species seem to be able to ingest and utilize phytoplankton^20,23,24^, small zooplankton with low mobility is the main component of the natural diet of many temperate species^22,25,26^. While considerable knowledge exists on the feeding biology and ecophysiology of shallow octocorals, such information is scarcer for deep-sea octocorals with a few studies so far focusing mainly on Antarctic ecosystems^20,27^.

**Figure 1:**
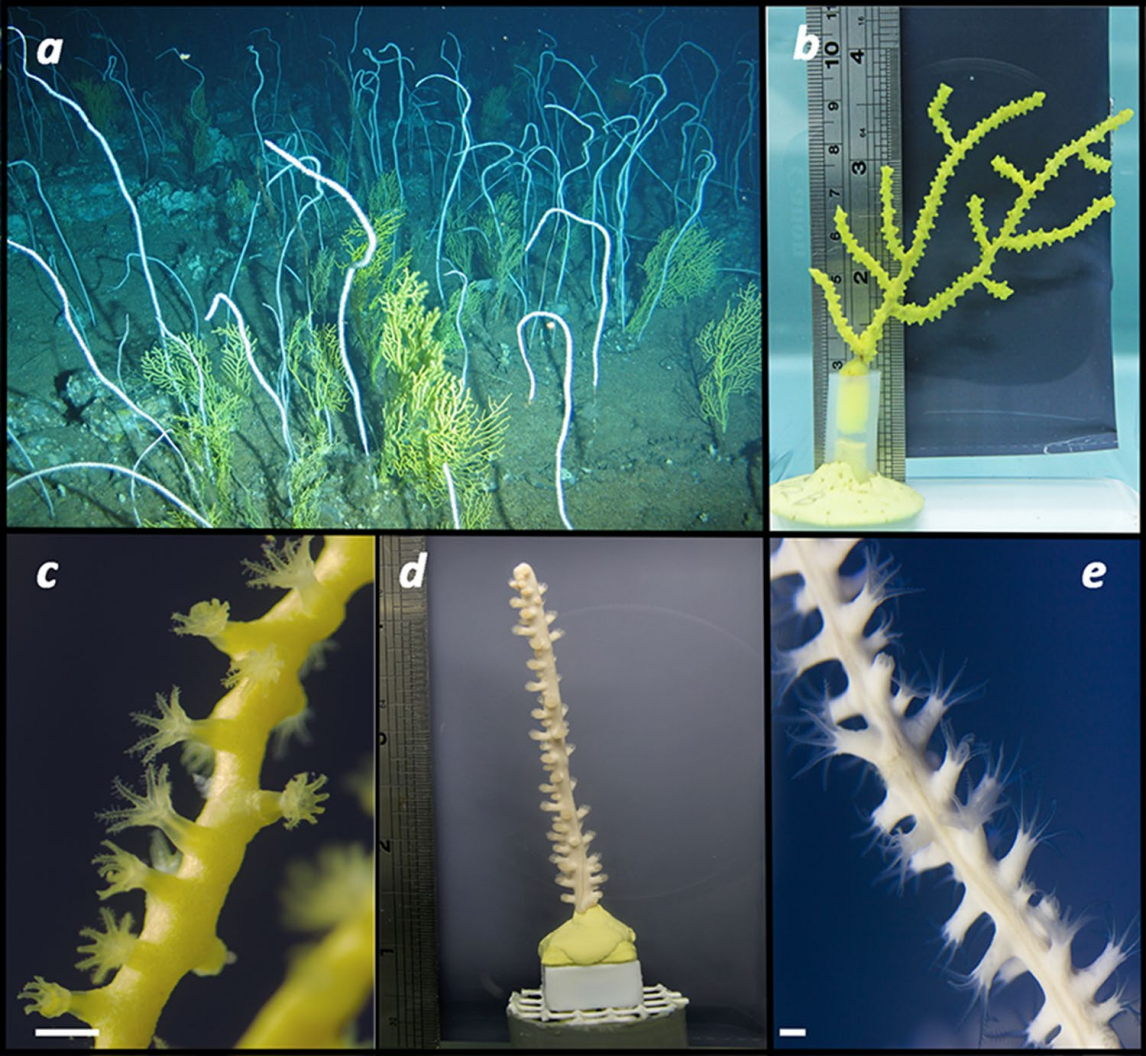
(**a**) Mixed coral garden of the octocorals *Viminella flagellum* and *Dentomuricea* aff*. meteor* (Gavin Newman, Greenpeace); coral fragment of *D.* aff*. meteor* (**b**) and its polyps (**c**); coral fragment of *V. flagellum* (**d**) and its polyps (**e**). Scale bar 1 mm.

In this study, we examined the feeding biology of two common habitat-forming deep octocoral species in the Azores Archipelago: *Dentomuricea* aff*. meteor* and *Viminella flagellum*. The two species form dense coral gardens (Fig. 1) on seamounts between 200–600 m and very frequently occur in mixed populations^7,9^. The objectives of the study were (1) to determine the ability of the two species to exploit different food sources and (2) to evaluate if assimilation of different food sources affects their metabolic activity. We employed an experimental approach, with the use of aquaria flumes with steady flow velocity and four different food treatments including provision of live phytoplankton, dissolved organic matter (DOM) and live zooplankton, as well as fasting (deprivation of particulate food). Food sources were artificially enriched with ^13^C and ^15^N and food utilization was quantified (a) as the appearance of ^13^C^15^N in the coral tissue, indicating tracer carbon (C) and nitrogen (N) incorporation from the provided food, (b) as the production of ^13^C-enriched dissolved inorganic carbon (DIC) by the coral, indicating tracer C respiration, and (c) as the production of ^13^C^15^N-enriched particulate organic carbon and nitrogen (POC, PON) by the coral, indicating tracer POC and PON release.

## Results

All utilized food sources were significantly enriched above background, i.e. above non-labelled food (Table 1). Target C concentrations within the aquaria were successfully achieved in the case of the DOM and ZOO treatment, however they were below the target for the PHYTO treatment, by 34% for *D.* aff*. meteor* and 44% for *V. flagellum* respectively (Table 1).

**Table 1:** Characteristics of enriched food sources used in feeding experiments of *Dentomuricea* aff*. meteor* and *Viminella flagellum*, including average ± SD of the created C concentrations in the aquaria, total provided C per coral fragment and per coral organic carbon, fractional abundance (F_13_, F_15_) and carbon to nitrogen (C/N) ratio. CC: *Chaetoceros calcitrans*, NG: *Nannochloropsis gaditana*, BP: *Branchionus plicatilis*.

Food source	*D.* aff*. meteor*				*V.flagellum*			
	*CC* (PHYTO)	*NG*	DOM	*BP* (ZOO)	*CC* (PHYTO)	*NG*	DOM	*BP* (ZOO)
C Concentration (μmol L^−1^)	7.28 ± 0.2	–	9.19	11.24 ± 0.5	5.59 ± 0.2	–	9.19	10.06 ± 0.5
Total provided C (μmol coral fragment^−1^)	287.93 ± 8.2	–	364.20	445.41 ± 22	369.09 ± 18.9	–	607.0	664 ± 33
Total provided C (μmol mmol coral C^−1^)	150.75 ± 5	–	190.68	233.94 ± 13	54.51 ± 2.7	–	89.66	98.14 ± 4.8
F_13_	0.58	0.49	0.96	0.21	0.59	0.58	0.96	0.37
F_15_	0.42	0.31	0.88	0.12	0.41	0.30	0.88	0.15
C/N	11.86	9.94	4.41	5.44	9.95	10.83	4.41	5.62

**Figure 2:**
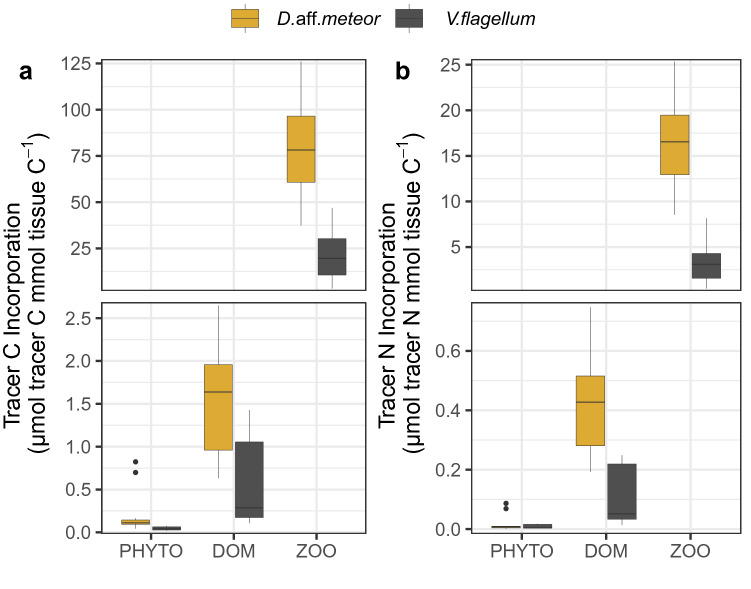
Tracer C (**a**) and N (**b**) incorporation (average ± SD) of the octocoral species *Dentomuricea* aff*. meteor* and *Viminella flagellum* upon provision of different food sources enriched with ^13^C and ^15^N. Axis breaks are used to highlight the large differences of tracer among treatments. PHYTO: phytoplankton; *Chaetoceros calcitrans*; DOM: dissolved organic matter; ZOO: zooplankton *Branchionus plicatilis.*

Coral fragments incorporated tracer C and N from all food sources in their tissue (Fig. 2). All results on tracers are presented as average ± standard deviation. Both species incorporated significantly higher tracer C and N under the ZOO treatment followed by the DOC treatment (Fig. 2, Table 2). Coral fragments of *D.* aff. *meteor* and *V. flagellum* under the ZOO treatment incorporated 422% and 453% more tracer C than under the PHYTO treatment, respectively. *Viminella flagellum* displayed lower tracer incorporation compared to *D.* aff*. meteor* in all treatments, reaching on average 64% lower C incorporation and 70% lower N incorporation than *D.* aff*. meteor* (Fig. 2). Total tracer C incorporation varied between 0.19 ± 0.24 μmol tracer C mmol tissue C^−1^ under the PHYTO treatment to 80.2 ± 24.5 μmol tracer C mmol tissue C^−1^ under the ZOO treatment for *D.* aff*. meteor* and from 0.05 ± 0.02 μmol tracer C mmol tissue C^−1^ under the PHYTO treatment to 21.8 ± 13.9 μmol tracer C mmol tissue C^−1^ under the ZOO treatment for *V. flagellum*.

**Table 2:** Coefficients of constructed models to explore the effect of four food treatments (FAST: fasting; PHYTO: diatom *Chaetoceros calcitrans*; DOM: dissolved organic matter; ZOO: rotifer *Branchionus plicatilis*) on each dependent variable after analysis of collected data on tissue, respiration and excretion of two octocoral species *Dentomuricea* aff*. meteor* and *Viminella flagellum*. If food source was excluded from the respective model during model construction, it was assumed it had no significant effect on the response variable in question and therefore no coefficients are provided. SE: Standard error.

Species		*Dentomuricea aff. meteor*							*Viminella flagellum*					
Variable group	Dependent variable	Treatment	Fixed effects			Random effects		Variance Structure	Fixed effects			Random effects		Variance Structure
			Value	SE	*p* value	Colony	Residual		Value	SE	*p* value	Colony	Residual	
Tissue	Tracer C Incorporation	PHYTO	0.18	0.091	0.040	0.172	0.602	0.27	0.05	0.006	0.000			1.00
		DOM	1.61	0.166	0.000			1.00	0.54	0.140	0.002			23.70
		ZOO	80.26	6.359	0.000			39.49	21.80	4.030	0.000			655.10
	Tracer N Incorporation	PHYTO	0.01	0.009	0.130	0.018	0.152	0.12	0.01	0.004	0.001			1.00
		DOM	0.40	0.041	0.000			1.00	0.12	0.126	0.001			16.30
		ZOO	16.66	1.159	0.000			28.45	3.36	0.447	0.000			355.90
Respiration	Oxygen consumption	FAST	1.08	0.199	0.001	0.070	0.430		0.11	0.047	0.040	0.080	0.090	
		PHYTO	0.98	0.290	0.716				0.23	0.050	0.278			
		DOM	1.61	0.290	0.089				0.14	0.050	0.514			
		ZOO	1.93	0.290	0.011				0.25	0.050	0.012			
	DIC Bulk respiration	FAST							0.24	3.090	0.017			
		PHYTO							0.18	− 0.540	0.605			
		DOM							0.26	0.140	0.887			
		ZOO												
	Tracer C respiration	PHYTO												
		DOM	0.00	0.001	0.002				0.00	3.770	0.013			0.06
		ZOO	0.23	0.055	0.003				0.06	3.110	0.264			1.00
POC/PON release	POC bulk release	FAST	0.03	0.013	0.062			1.00						
		PHYTO	0.07	0.026	0.207			1.06						
		DOM	0.09	0.015	0.002			0.45						
		ZOO	0.21	0.060	0.002			3.78						
	Tracer C release	PHYTO	0.00	0.002	0.104			1.00	0.00	0.000	0.060			2.49
		DOM	0.00	0.001	0.440			0.69	0.00	0.000	0.419			1.00
		ZOO	0.10	0.029	0.003			20.92	0.01	0.001	0.000			9.49
	PON bulk release	FAST	0.01	0.011	0.240									
		PHYTO	0.07	0.014	0.728									
		DOM	0.04	0.014	0.054									
		ZOO	0.03	0.014	0.192									
	Tracer N release	PHYTO	0.00	0.000	0.000			1.00	0.00	0.000	0.000			0.18
		DOM	0.00	0.001	0.050			13.50	0.00	0.000	0.532			1.00
		ZOO	0.02	0.004	0.000			102.70	0.00	0.001	0.003			3.06

**Figure 3:**
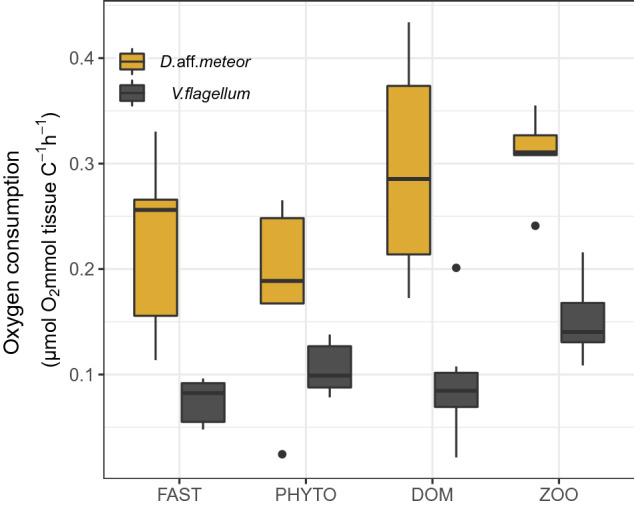
Oxygen consumption (average ± SD) of the octocoral species *Dentomuricea* aff*. meteor* and *Viminella flagellum* upon provision of different food sources. Coral fragments were fed with the respective food source for four days and oxygen was measured in closed-cell incubations that took place immediately after feeding on day four and lasted for approximately 12-14 h. FAST: no food provision; PHYTO: phytoplankton *Chaetoceros calcitrans*; ZOO: zooplankton *Branchionus plicatilis*; DOM: dissolved organic matter.

In both species, oxygen consumption was significantly higher in the ZOO treatment compared to the other food treatments (Table 2), reaching on average 0.308 ± 0.042 μmol Ο_2_ mmol tissue C^−1^ h^−1^ for *D.* aff*. meteor* and 0.151 ± 0.036 μmol Ο_2_ mmol tissue C^−1^ h^−1^ for *V. flagellum* (Fig. 3). Overall, oxygen consumption was almost two times higher in fragments of *D.* aff*. meteor* compared to *V. flagellum*.

**Figure 4:**
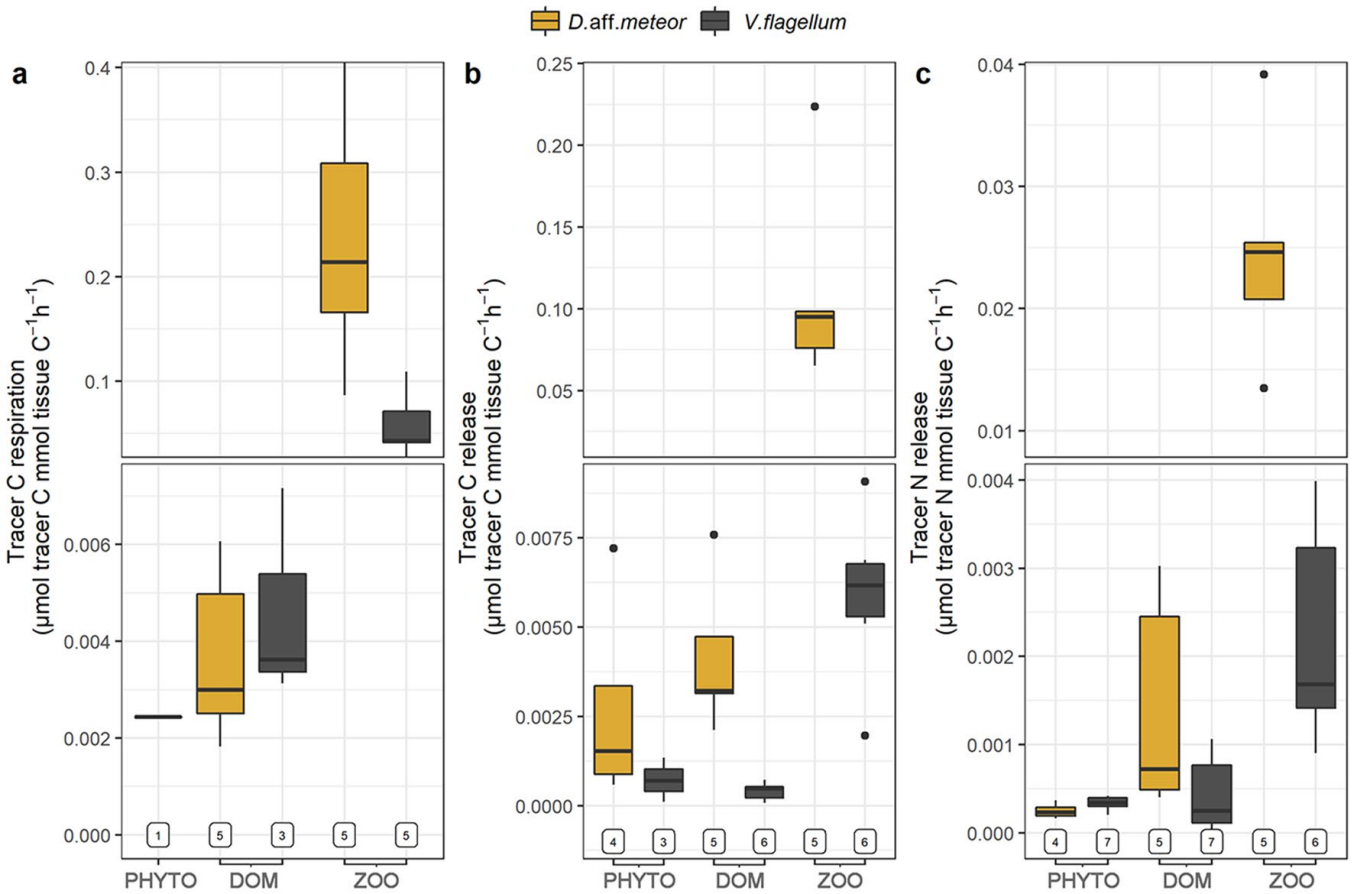
Tracer fluxes (average ± SD), including tracer C respiration (**a**), tracer C release (**b**) and tracer N release (**c**) of the octocoral species *Dentomuricea* aff*. meteor and Viminella flagellum* upon provision of different food sources. Numbers below bars represent the number of coral fragments for which positive estimates were obtained (max 7). Axis breaks are used to highlight large differences in scale among some treatments. PHYTO: phytoplankton *Chaetoceros calcitrans*; DOM: dissolved organic matter; ZOO: zooplankton *Branchionus plicatilis*.

Both species utilized tracer C and N derived from the provided food for C respiration, POC and PON release (Fig. 4). Tracer C respiration was significantly higher under the ZOO treatment for both species (Fig. 4, Table 2). Similarly, for both species tracer POC and PON release were higher under the ZOO treatment while fragments under the DOM and PHYTO treatments displayed very low average values of POC and PON release, which did not differ significantly from zero (Table 2).

**Figure 5:**
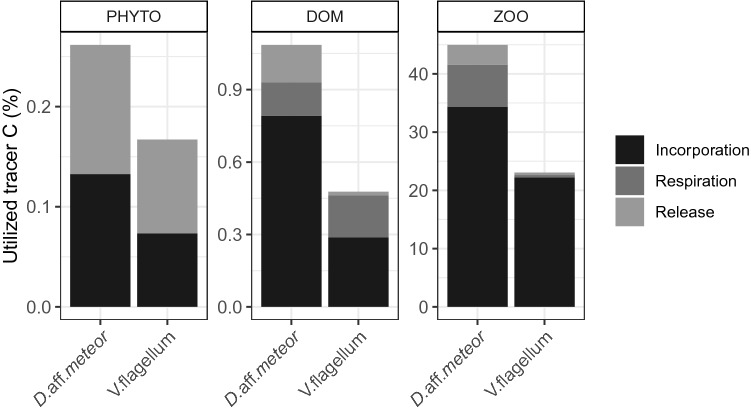
Tracer utilized by fragments of two octocoral species *Dentomuricea* aff*. meteor* and *Viminella flagellum*, expressed as a percentage of the provided carbon of different food sources: PHYTO: phytoplankton *Chaetoceros calcitrans*; DOM: dissolved organic matter; ZOO: zooplankton *Branchionus plicatilis*.

To take into account differences in the provided C quantity, the amount of utilized C tracer is provided as a percentage of the total provided C in Fig. 5. For *D.* aff*. meteor* 45% of the provided zooplankton-derived tracer C could be traced back in tissue incorporation, DIC respiration and POC release, with phytoplankton-derived and DOM-derived tracer C reaching 1.08% and 0.26%, respectively (Fig. 5). Under the PHYTO treatment, corals utilized most of the C tracer for POC release, while in the two other treatments they utilized most of the C tracer for tissue incorporation, followed by tracer C respiration (Fig. 5). In *V. flagellum,* a smaller percentage of the provided C was traced back, reaching 23% under the ZOO treatment, 0.48% under the DOM treatment and 0.17% under the PHYTO treatment.

## Discussion

To our knowledge this is the first study addressing the feeding biology of deep octocoral species by employing an experimental approach with the use of stable isotopes. The strong differences in tracer C and N incorporation among the food treatments suggest a higher efficiency in capture and ingestion of zooplankton prey when this resource is available, compared to the other food treatments. Previous studies have highlighted zooplankton as an important dietary component of deep-sea scleractinians^28–31^. However, only a few deep-sea octocorals, mainly of the family Primnoidae, have been shown to base their diet on microzooplankton^32,33^ while most studied species rely mostly on phytodetritus and particulate organic matter (POM)^28,34^. Our results provide a direct demonstration of the importance of zooplankton for some deep-sea octocorals and highlight that they might be more selective than previously thought.

In areas with strong hydrodynamics, such as seamounts, fresh phytoplankton can be directly transported to great depths by rapid downwelling and tidal waves^35,36^, and therefore it might be often available for the two target octocorals. The closely positioned pinnules of octocoral tentacles and their relatively weak nematocysts generally suggest herbivory^24,37,38^. Moreover, in previous studies, both target octocorals have been shown to occupy low trophic levels, placed between primary and secondary consumers^39^. Thus, the lower incorporation of phytoplankton was unexpected. It is possible that during the experiment, coral fragments fed additionally on small particles (< 1 μm) that passed the filtration system. This could explain the lower DOM and phytoplankton utilization, but it further supports the hypothesis that both species can display selective feeding.

The feeding behavior of the two target octocorals is in strong contrast to the results of similar feeding experiments with the deep-sea scleractinian *Lophelia pertusa* (recently synonymized to *Desmophyllum pertusum*^40^) which displayed a rather unselective feeding behavior under feeding with similar food sources and flow velocities^41^. These differences might be due to the use of processed (freeze-dried) versus live prey. The use of isotopically enriched live prey has been used before to study the feeding preferences of octocorals^42,43^ and scleractinians^44,45^. It allows more realistic comparisons compared to dry food, as it takes into consideration both the capture and ingestion ability of the study species. On the other hand, it includes a considerable error in determining and standardizing provided C quantities since C content can vary among culture batches. In the present study, the available C in the aquaria of the DOM and ZOO treatments was 30–40% higher than in the PHYTO treatment, thus a proportionately higher utilization of the DOM and ZOO food sources was expected. While this can explain the small differences in tracer C utilization between the PHYTO and DOM treatments, it cannot explain the disproportionally larger tracer utilization under the ZOO treatment, strongly indicating more efficient feeding on zooplankton.

Since zooplankton dynamics in the study area follow seasonal phytoplankton productivity cycles^46^, it is likely that this important food source displays strong seasonal fluctuations. Both target species displayed the ability to utilize food sources variable in size and composition, therefore during the rest of the year it is very likely that the two species sustain their metabolism through feeding on other sources such as phytoplankton and DOM. Dissolved organic matter has proven to be an important food source for cold-water scleractinians when particulate food sources are scarce^47^. The ability to utilize DOM can be very important in oligotrophic deep-sea environments where food availability is seasonal and is likely to be further affected by climate change^48^. A number of previous studies have highlighted the variable and seasonally-dependent diet of octocoral species in temperate ecosystems^19,49,50^ while similar seasonality has been also reported in benthic Antarctic ecosystems^27,51^.

The two target octocorals displayed higher oxygen consumption and tracer C respiration upon feeding with zooplankton, highlighting the importance of zooplankton to meet their respiratory and metabolic demands. Similar results have been reported for *Desmophyllum dianthus* which displayed lower oxygen respiration, calcification and TOC release after exclusion of zooplankton from available food sources^31^. Because of the seasonal availability of zooplankton and its importance for tissue incorporation and metabolism, it appears likely that physiological processes that require the development of C and N rich tissues, such as growth and reproduction, may also undergo strong seasonality. This is a common phenomenon for octocorals in temperate areas, which display seasonal cycles in their biochemical levels e.g.^52^ and often pass through periods of metabolic dormancy in summer months when available seston is scarce and temperature rises^16,53^. D*entomuricea* aff*. meteor* and *V. flagellum* display gamete presence all year round with frequent seasonal peaks in spring and autumn (M. Rakka, unpubl.), which could be related to higher zooplankton availability^46^, but more studies on abiotic conditions and physiological cycles are essential to unravel their ecophysiology.

Fragments fed with zooplankton also displayed higher tracer POC and PON release in both species, which in corals is associated with mucus production, essential in processes such as feeding, cleansing and protection from epibionts and pathogens^54^. Mucus production can be extremely important to protect the corals against mechanical and chemical disturbance due to bottom trawling, oil extraction and mining (e.g. mine tailing and drill cutting resuspension)^55–57^. Moreover, coral mucus has been identified as an ecologically important element for CWC communities, since it enhances microbial activity and therefore mineralization, recycling and overall ecosystem productivity^54,58^. The increased POC release under the ZOO treatment showcases how feeding on zooplankton can enhance the contribution of octocoral species to C recycling and highlights their importance for bentho-pelagic coupling.

Overall, *D.* aff*. meteor* appeared to acquire higher percentage of the provided C compared to *V. flagellum*. Moreover, when fed with the most effectively utilized food source (zooplankton), *D.* aff*. meteor* allocated a higher proportion of captured C to respiration and POC release compared to *V. flagellum*. This highlights the different strategies adopted by the two species. While *V. flagellum* appeared more conservative in resource allocation, storing most of the captured C in tissue, and minimizing losses, the pattern displayed by *D.* aff*. meteor* is indicative of “sloppy feeding”, in which high amounts of the captured C are lost during the feeding process^59,60^. Feeding behaviour and metabolic rates can be influenced by an array of factors, such as environmental variables, physiological status and life stage. The two target species were haphazardly collected, maintained in similar conditions and were expected to be in similar stages in their reproductive cycle (M. Rakka, unpubl.). Thus, variations in colony health, age and maturity are more likely to explain the observed variance within each species, while the marked difference between the two octocorals may be attributed to species-specific characteristics, such as morphology and growth pattern^61^. The species *D.* aff*. meteor* has a branching pattern with high surface to volume ratio and possesses a large number of small polyps which can increase both capture rates and metabolic costs^14,62^. On the other hand, *V. flagellum* displays an erect growing pattern with bigger polyps and a lower surface to volume ratio that may have lower maintenance costs.

Both target species incorporated a lower amount of tracer from phytoplankton compared to *L. pertusa*^63^. In contrast, fragments fed with zooplankton displayed higher tracer incorporation than *L. pertusa*, i.e. 10 times higher for *D.* aff*. meteor* and two times higher for *V. flagellum*. Although these differences might be attributed to the use of dry versus live prey, they highlight that resource acquisition strategies are species-specific, as also demonstrated for several tropical and cold-water coral (CWC) species^18,28,33^. Metabolism is tightly connected to the ecological niche of a species, and different responses to food supply can explain distributions of species and species assemblages^64^. For example, *D.* aff*. meteor*, due to its metabolic strategy presented herein, is expected to have an advantage under high food concentration, however, it is unlikely to outperform *V. flagellum* under low food conditions.

Similarly to reefs formed by cold-water scleractinian species^65,66^, the rich organic excretion of octocorals promotes organic cycling and plays an important role supporting a diverse community of associated fauna in coral gardens and adjacent deep-sea communities^67^. Taking into account that the two species displayed different strategies in respect to the respired and released C, we hypothesize that the communities dominated by *D.* aff*. meteor* are likely to be characterized by higher C and N recycling whereas communities of *V. flagellum* will likely have higher residence time of C and N in the coral tissue, with further consequences for the local C cycle. Sloppy feeding is known as an important behavior for the support of C cycles, fueling the microbial loop and supporting local food webs^68,69^. Thus, the role of these species to local and global marine biogeochemical cycles should be further investigated.

In conclusion, the present study provides important knowledge on the resource utilization and metabolic strategies of two important habitat forming octocorals, that can help understand patterns at the species, population and community level^70^. Species distribution modelling has shed light to the distribution of different deep-sea coral groups, including octocorals^71^, but comparatively little effort has been made to delve further into the biological and physiological characteristics which shape these distributions^72,73^. Taking into account these traits will not only improve predictions on species occurrences and help to identify priority areas for conservation and management^74^, but will also provide a more robust understanding of the ecology of deep-sea corals. Coral resource use and metabolism is likely to change under future conditions of increased seawater temperature, stratification of water masses and consequent reduction in the quantity and quality of POM flux to the seafloor^48^. Further studies on the ecophysiology of octocoral species under present and future scenarios of climate change are therefore essential to improve our understanding of the distribution and ecological function of deep-sea communities.

## Methods

### Target species

The species *Dentomuricea* aff*. meteor* is a fan-shaped alcyonacean of the family Plexauridae. Its known distribution is limited to seamounts close to the Mid-Atlantic ridge where it is typically encountered between 200 and 400 m depth^7^. It can reach heights of up to 1.5 m and can create dense monospecific or mixed populations with other species (Fig. 1a).

*Viminella flagellum*^75^ is a whip coral of the family Ellisellidae. It creates monopodial colonies without branches which can grow up to 3 m height^76^. Its distribution includes the eastern North Atlantic coast, islands of the Macaronesia and the Mediterranean Sea^77–79^. It is usually encountered between 120 and 500 m depth^77,78^ and can form monospecific or mixed coral aggregations, often with branching octocorals such as *D.* aff*. meteor*, *Callogorgia verticillata and Acanthogorgia armata*^80^.

### Colony collection and maintenance

Live colonies of both *V. flagellum* and *D.* aff*. meteor* were collected as by-catch from long-line fisheries on R/V *Archipelago* (ARQDAÇO monitoring program, University of the Azores) and on commercial fishing vessels through a fisheries observer program, during September–November 2017. Collection was performed in various seamounts within the Azorean EEZ (Supplementary Table S1). Colonies were transferred to the aquaria facilities of IMAR (DeepSeaLab) in coolers and distributed in three 170 L aquaria in a thermo-regulated room, in darkness. Corals were inspected for potential tissue injuries from the collection process and colonies with unhealthy tissue were discarded. Aquaria were supplied with seawater pumped from 5 m depth in continuous flow-through open systems. Before entering the aquaria, water was treated with UV-light (P10 UVsystem and Vecton 600, TMC) and was repeatedly filtered (mesh size: 50 μm and 1 μm). Temperature was maintained at 14 ± 0.7 °C, which is similar to the temperature recorded at coral gardens of the two species ^81^, by cooling systems connected to temperature controllers. Corals were fed daily with a frozen mixture of microalgae, microzooplankton and frozen thawed macrozooplankton (*Artemia* nauplii, *Mysis* shrimps), which was enriched with live microalgae and rotifers 2–3 times per week. The collected colonies were left to acclimatize for approximately three months in the aforementioned conditions. Subsequently, colonies were divided in 8–10 cm fragments and mounted to bases made of epoxy one month before the experiments (Fig. 1b,d). During this period, fragments were closely monitored to ensure that they had vibrant colour, intact tissue, and displayed polyp activity.

### Feeding experiment

Four different food treatments were created, based on: a phytoplankton derived source (PHYTO), a zooplankton derived source (ZOO), a dissolved organic carbon (DOM) derived source and a fourth treatment where no particulate food source (size > 1 μm) was provided (fasting, FAST). Food treatments were created based on current knowledge of the species biology and on available food sources in their natural environment. The diatom species *Chaetoceros calcitrans* was selected as a phytoplankton-derived food source. Species of the genus *Chaetoceros* are common components of spring blooms in some of the sampling sites, e.g. Condor Seamount^81^. The rotifer *Branchionus plicatilis* was selected as zooplankton-derived food source due to its small size (140–330 μm) and slow swimming capacity, which correspond to the characteristics of zooplankton prey usually captured by octocoral species^22,26^. Due to the known capacity of cnidarians to utilize DOM^47,82^ this food source was also used as a food treatment. Lastly, the absence of additional food (FAST) aimed at measuring the basal metabolic activity of the corals, in the absence of particulate food.

**Figure 6:**
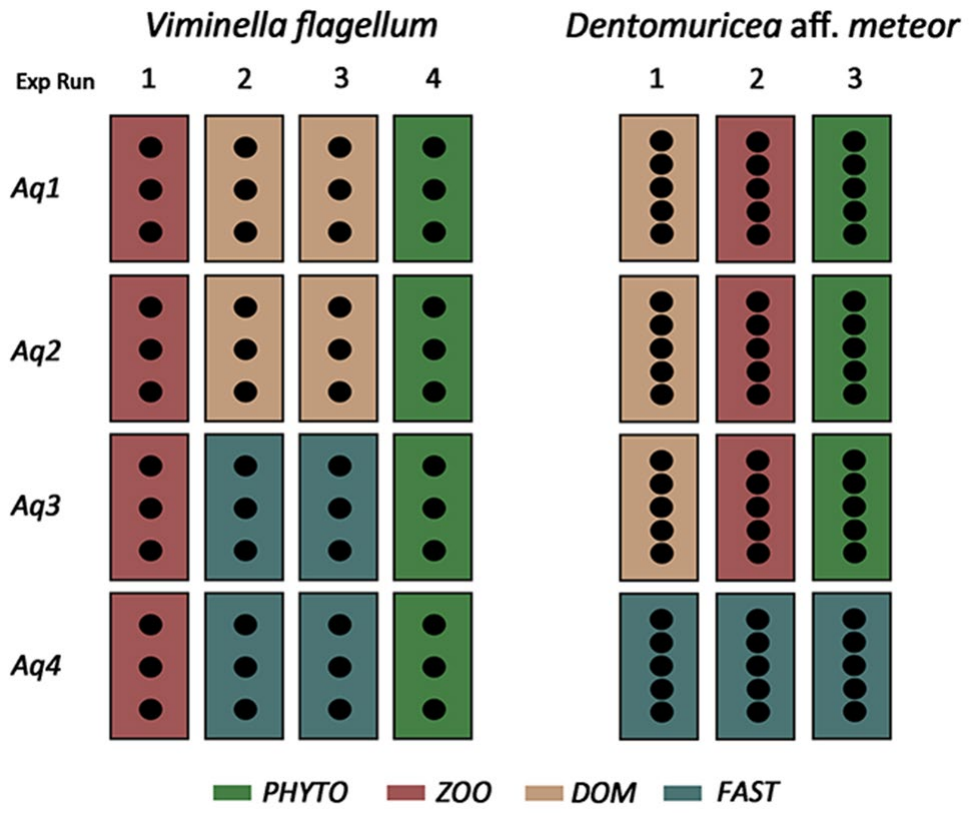
Experimental design of the two feeding experiments with the octocoral species *Dentomuricea* aff*. meteor* and *Viminella flagellum*. Exp cycle: Experimental cycles; Aq: Aquaria; PHYTO: phytoplankton *Chaetoceros calcitrans*; ZOO: zooplankton *Branchionus plicatilis*; DOM: dissolved organic matter; FAST: no food provision. Rectangles represent experimental aquaria and black dots represent coral fragments.

The feeding experiment was run in four 33 L flumes designed to keep live prey in continuous circulation (Supplementary Fig. S1), which allowed the use of a multilevel experimental design (Fig. 6). The different number of available specimens for the two species led to a slightly different experimental design for each (Fig. 6), however the same rationale was followed for both species. One month before the experiment, each mother colony (n = 5 for *D.* aff*. meteor* and n = 12 for *V. flagellum*) was divided in smaller fragments (n = 5 for each colony of *D.* aff *meteor* and n = 4 for each colony of *V. flagellum*) and these were randomly distributed to the four food treatments. The characteristics of fragments in the different treatments are presented in Supplementary Table S2. Because of the limited number of experimental flumes, we repeated the experimental work several times, in order to have more than one aquaria replicates for each food treatment (n = 3 for *D.* aff*. meteor* and n = 4 for *V. flagellum*). Each repetition is referred to as experimental cycle (Fig. 6). At the beginning of each experimental cycle, coral fragments were randomly positioned in the available aquaria for each treatment. This led to a total of 15 coral fragments for each food treatment for the case of *D.* aff*. meteor* and 12 coral fragments for each food treatment for *V. flagellum*. Dependence among fragments on the colony and aquaria level was treated statistically, by the use of mixed effects models (see statistical analysis).

Before each experimental cycle, zooplankton and phytoplankton food sources were prepared by adding enhanced levels of the stable isotope tracers ^13^C and ^15^N to the respective culture media. Two microalgae species, *C. calcitrans* and *Nannochloropsis gaditana* were cultured using artificial seawater and an F/2 culture medium containing 50% ^15^N-sodium nitrate (NaNO_3_, Cambridge Isotopes) and 100% ^13^C-bicarbonate (NaHCO_4_, Cambridge Isotopes) for three weeks. Subsequently, cultures were harvested by filtering with membrane filters (0.2 μm), rinsed with filtered SW (0.2 μm) and re-suspended in artificial SW. Rotifer starter cultures (concentration: 45 rotifers ml^−1^) were inoculated in filtered seawater (1 μm), and continuously fed with ^13^C^15^N-enriched *N. gaditana*, cultured as described above, for 6 days. Rotifer cultures were harvested by filtering (nylon filters, 40 μm), rinsed and re-suspended in artificial seawater. Preliminary analysis was performed to ensure that harvesting procedures did not affect cell concentration significantly. An algal-derived product of dissolved amino-acids (Cambridge Isotopes, U 13C 97–99%, U ^15^N 97–99%, CNLM-452–0.5) was used as DOM food source.

Prey was provided to a target carbon (C) concentration of 10 μmol L^−1^, similar to POM concentrations previously recorded in mixed gardens of the two species (A. Colaço, pers. comm.). Cultures were scheduled to reach the desired prey concentration, corresponding to the desired target C concentration, on the day of delivery and were harvested a few hours before provision. To monitor the experimental food concentrations, aliquots of the provided food were taken before provision and analyzed a posteriori for DW and carbon content.

For both species, each experimental cycle consisted of five days. At the start of each cycle, fragments were positioned in the aquaria one next to the other, perpendicular to the current, avoiding branch overlapping. Once per day a predefined quantity of food was provided to reach a concentration of 10 μmol C L^−1^. Flow of 4 cm s^−1^ was established for one hour and water renewal was paused just before supplying aquaria with food. This flow speed was selected as it allowed both species to capture live prey (Rakka et al., unpublished data) and kept prey in suspension for 12 h without affecting its concentration. After 12 h, water renewal was reestablished and all remaining food was cleaned by siphoning.

In the last day of each experimental cycle and immediately after the end of feeding, closed cell incubations were performed to measure oxygen consumption, DIC respiration and POC/PON release. Seven coral fragments from each food treatment were transferred to 450 ml glass chambers with pre-filtered seawater (0.7 μm) and glass-coated magnetic stirrers. All chambers were placed in a water bath keeping temperature at 14 ± 0.5 °C. Another two chambers were left without coral fragments and served as controls. Respiration rates were derived from depletion of dissolved O_2_ during the incubation, measured by a single channel oxygen meter (Fibox4) with a PSt3 sensor (PreSens, Germany). The chamber with the larger fragment was continuously connected to the sensor to monitor oxygen saturation levels during the incubations. Each incubation lasted for approximately 14 h in which oxygen saturation never dropped below 80%. Oxygen consumption was standardized to the tissue organic carbon content (OC), i.e. without taking into account the main skeletal axis. These values were adjusted for rates recorded in chambers without coral fragments to account for microbial respiration. Water samples were taken before and after each incubation to determine the concentration of DIC and ^13^C-DIC. Samples were kept in 10 mL headspace vials with 10 μL of a saturated mercury chloride solution and stored at 4 °C until analysis. Lastly, the remaining water (300 ml) from each chamber after the end of the incubation was filtered through precombusted, preweighted GF/F (0.7 μm, WHATMAN) filters to estimate POC, ^13^C-POC, PON and ^15^N-PON release. Filters were freeze-dried and kept at room temperature until analysis.

Upon completion of each experimental cycle, fragments were freeze-dried and stored at -80 ºC. Fragments were dissected to separate the tissue from the skeleton. The tissue was ground by mortar and pestle, and a subsample was analyzed for total C and N content and isotopic ratios using an elemental analyzer (Thermo Electron Flash 1112) coupled to an isotope ratio mass spectrometer (EA‐IRMS, DELTA‐V, THERMO Electron Corporation). A second subsample was acidified stepwise with drops of HCl to remove the inorganic C fraction, and all remaining material was analyzed on the elemental analyzer for organic C content and isotopic ratio.

Calculation of tracer C and N incorporation, i.e. calculation of the amount of C and N which came from the provided food source and was incorporated in coral tissue, was performed as described in Maier et al.^63^. Tissue C and N content of each fragment (i.e. without coral skeleton) was standardized to DW, and expressed as mmol C or N (g DW)^−1^. The heavy/light isotope ratio (e.g. ^13^C: ^12^C) of each coral fragment (Rsample) was calculated as Rsample = ([δtracerCsample/1000] + 1) × Rref, where Rref = 0.0111802 for organic C (OC) and Rref = RN_2_ = 0.0036782 for organic N (ON). Fractional abundance of ^13^C and ^15^N (e.g. F^13^ = ^13^C/[^12^C + ^13^C]) was expressed as Ftracer = Rsample/(Rsample + 1). Experimental ^13^C and ^15^N enrichment of each coral fragment tissue was expressed in relation to the fractional abundance of the respective fasting (non-enriched) fragment or average of fasting fragments of the same colony. Finally, tracer ^13^C incorporation was calculated by multiplying ^13^C enrichment with tissue OC content (μmol ^13^C fragment^−1^) and tracer ^15^N incorporation was obtained by multiplying ^15^N enrichment with tissue ON content (μmol ^15^N fragment^−1^). The total amount of C or N incorporated into coral tissue from the provided labelled food source (tracer C and N incorporation) was calculated by dividing the tracer C or N incorporation of each fragment with the fractional abundance (F^13^ or F^15^) of the respective food source. Final tracer C and N incorporation rates were normalized to the OC (mmol) of each coral fragment.

Concentration of DIC was determined on an Apollo SciTech AS-C3 analyzer, after transforming DIC to gaseous carbon dioxide by addition of concentrated phosphoric acid (H_3_PO_4_, volume: 10 μL mL^−1^) in each headspace vial. Subsequently, a 10 μL subsample of headspace gas was obtained from each vial and analyzed in the isotope ratio mass spectrometer, as described above, to obtain measurements of δ^13^C. Filters collected for POC and PON measurements were weighted (accuracy 0.1 mg) and analyzed with the isotope ratio mass spectrometer to obtain concentrations of POC and PON, as well as δ^13^C of and δ^15^N respectively. Determination of fluxes, i.e. DIC respiration, POC and PON release was estimated in two steps. Firstly, the bulk fluxes, i.e. the amount of total C and N released during the incubation period were calculated as the respective concentration difference between start and end water sample. Subsequently, tracer fluxes, i.e. the amount of C and N derived from the provided food were estimated, by multiplying bulk fluxes by their relative enrichment in ^13^C and ^15^N during the incubation, and dividing by the food enrichment following Maier et al.^83^. A final tracer C budget was compiled by estimating tracer C incorporation, tracer C respiration and tracer C release for the duration of the whole experiment for each treatment and is reported as percentage of the provided C.

### Statistical analysis

Data exploration was done following Zuur et al.^84^ to select the most appropriate statistical modelling method. To test if independent factors had a significant effect on the dependent variables in question, the former were added progressively to the models and the Akaike Information Criterion (AIC) along with maximum likelihood ratio (MLR) tests were used to select the most appropriate model. Model diagnostics were inspected to detect potential violation of model assumptions. Statistical analysis was performed in R 3.5.0 (R Core Team, 2018). We provide detailed results of the MLR tests in the supplementary information (Supplementary Table S3) and coefficients of the best models in Table 2.

Linear Mixed Effects Models (LMEs) and GLSs were used to analyze all response variables. Colony and experimental aquaria were incorporated as crossed random factors to deal with dependence related with: (1) the existence of multiple coral fragments that originated from the same colony and (2) the fact that multiple coral fragments were positioned in the same aquaria. Whenever the assumption of homogeneity of variance was not fulfilled, variance structure components were added to the models to allow the variance to differ among tested treatments^85^. LME models were build using the packages *LME4*^86^ and *nlme*^87^.

## Data availability

The datasets generated and analyzed in the current study are available in the Pangaea repository, under the following link: https://doi.pangaea.de/10.1594/PANGAEA.913184.

## Supplementary Information

Supplementary Information.

## References

[1] Watling L, France SC, Pante E, Simpson A (2011). Biology of Deep-Water Octocorals. Advances in Marine Biology.

[2] Sánchez, J. A. Diversity and Evolution of Octocoral Animal Forests at Both Sides of Tropical America. in *Marine Animal Forests *(ed. Rossi, S., Bramanti, L., Gori, A., & Orejas, C) 1–33 (Springer, 2016).

[3] Rossi, S., Bramanti, L., Gori, A. and Orejas, C. *Marine animal forests: the ecology of benthic biodiversity hotspots.* 1-1366. (Springer International Publishing, 2017)

[4] Cairns, S. D. Studies on western Atlantic Octocorallia (Gorgonacea: Primnoidae). Part 8: New records of Primnoidae from the New England and Corner Rise Seamounts. *Proceedings of the Biological Society of Washington* **120**(2), 243–263 (2007).

[5] Freiwald, A. and Roberts, J.M. *Cold-water corals and ecosystems*. (Springer, 2005)

[6] Buhl-Mortensen, L. & Buhl-Mortensen, P. Cold Temperate Coral Habitats. in *Corals in a Changing World* (2018).

[7] Braga-Henriques A (2013). Diversity, distribution and spatial structure of the cold-water coral fauna of the Azores (NE Atlantic). Biogeosciences.

[8] Íris S, Andre F, Filipe MP, Gui M, Marina C-S (2019). Census of Octocorallia (Cnidaria: Anthozoa) of the Azores (NE Atlantic) with a nomenclature update. Zootaxa.

[9] Tempera F (2012). Mapping condor seamount seafloor environment and associated biological assemblages (Azores, NE Atlantic). Seafloor Geomorphol. Benthic Habitat.

[10] Andrews A, Stone R, Lundstrom C, DeVogelaere A (2009). Growth rate and age determination of bamboo corals from the northeastern Pacific Ocean using refined 210Pb dating. Mar. Ecol. Prog. Ser..

[11] Neves BDM, Edinger E, Layne GD, Wareham VE (2015). Decadal longevity and slow growth rates in the deep-water sea pen Halipteris finmarchica (Sars, 1851) (Octocorallia: Pennatulacea): implications for vulnerability and recovery from anthropogenic disturbance. Hydrobiologia.

[12] FAO. *International guidelines for the management of deep-sea fisheries in the High Seas.* (2009).

[13] OSPAR. *Background document for coral gardens, Biodiversity Series, Publication Number: 15486/2010*. (2010).

[14] Kim K, Lasker HR (1998). Allometry of resource capture in colonial cnidarians and constraints on modular growth. Funct. Ecol..

[15] Gori A (2013). Effects of food availability on the sexual reproduction and biochemical composition of the Mediterranean gorgonian Paramuricea clavata. J. Exp. Mar. Bio. Ecol..

[16] Coma R, Ribes M (2003). Seasonal energetic constraints in Mediterranean benthic suspension feeders: effects at different levels of ecological organization. Oikos.

[17] Nisbet RM, Muller EB, Lika K, Kooijman SALM (2008). From molecules to ecosystems through dynamic energy budget models. J. Anim. Ecol..

[18] Sebens, K., Sarà, G. & Nishizaki, M. Energetics, Particle Capture, and Growth Dynamics of Benthic Suspension Feeders. in *Marine Animal Forests* 813–854 (Springer, 2017).

[19] Ribes M, Coma R, Gili JM (1999). Heterogeneous feeding in benthic suspension feeders: The natural diet and grazing rate of the temperate gorgonian Paramuricea clavata (Cnidaria: Octocorallia) over a year cycle. Mar. Ecol. Prog. Ser..

[20] Orejas C, Gili JM, Arntz W (2003). Role of small-plankton communities in the diet of two Antarctic octocorals (Primnoisis antarctica and Primnoella sp.). Mar. Ecol. Prog. Ser..

[21] Ribes M, Coma R, Rossi S (2003). Natural feeding of the temperate asymbiotic octocoral-gorgonian Leptogorgia sarmentosa (Cnidaria: Octocorallia). Mar. Ecol. Prog. Ser..

[22] Cocito S (2013). Nutrient acquisition in four Mediterranean gorgonian species. Mar. Ecol. Prog. Ser..

[23] Leal MC (2014). Temporal changes in the trophic ecology of the asymbiotic gorgonian Leptogorgia virgulata. Mar. Biol..

[24] Fabricius KE, Benayahu Y, Genin A (1995). Herbivory in Asymbiotic Soft Corals. Science (80-).

[25] Rossi S, Ribes M, Coma R, Gili JM (2004). Temporal variability in Zooplankton prey capture rate of the passive suspension feeder Leptogorgia sarmentosa (Cnidaria: Octocorallia), a case study. Mar. Biol..

[26] Coma R, Llorente-Llurba E, Serrano E, Gili JM, Ribes M (2015). Natural heterotrophic feeding by a temperate octocoral with symbiotic zooxanthellae: a contribution to understanding the mechanisms of die-off events. Coral Reefs.

[27] Orejas C, Gili J, López-González P, Arntz W (2001). Feeding strategies and diet composition of four Antarctic cnidarian species. Polar Biol..

[28] Sherwood OA, Jamieson RE, Edinger EN, Wareham VE (2008). Stable C and N isotopic composition of cold-water corals from the Newfoundland and Labrador continental slope: Examination of trophic, depth and spatial effects. Deep. Res. Part I Oceanogr. Res. Pap..

[29] Kiriakoulakis, K. *et al.* Lipids and nitrogen isotopes of two deep-water corals from the North-East Atlantic: initial results and implications for their nutrition. in *Cold-Water Corals and Ecosystems* 715–729 (Springer, 2005).

[30] Naumann MS, Tolosa I, Taviani M, Grover R, Ferrier-Pagès C (2015). Trophic ecology of two cold-water coral species from the Mediterranean Sea revealed by lipid biomarkers and compound-specific isotope analyses. Coral Reefs.

[31] Naumann MS, Orejas C, Wild C, Ferrier-Pagès C (2011). First evidence for zooplankton feeding sustaining key physiological processes in a scleractinian cold-water coral. J. Exp. Biol..

[32] Sherwood O (2005). Stable isotopic composition of deep-sea gorgonian corals Primnoa spp.: a new archive of surface processes. Mar. Ecol. Prog. Ser..

[33] Imbs AB, Demidkova DA, Dautova TN (2016). Lipids and fatty acids of cold-water soft corals and hydrocorals: a comparison with tropical species and implications for coral nutrition. Mar. Biol..

[34] Salvo F, Hamoutene D, Hayes VEW, Edinger EN, Parrish CC (2018). Investigation of trophic ecology in Newfoundland cold-water deep-sea corals using lipid class and fatty acid analyses. Coral Reefs.

[35] Davies AJ (2009). Downwelling and deep-water bottom currents as food supply mechanisms to the cold-water coral Lophelia pertusa (Scleractinia) at the Mingulay Reef Complex. Limnol. Oceanogr..

[36] Agusti S (2015). Ubiquitous healthy diatoms in the deep sea confirm deep carbon injection by the biological pump. Nat. Commun..

[37] Fabricius KE, Genin A, Benayahu Y (1995). Flow-dependent herbivory and growth in zoxanthellae-free soft corals. Limnol. Oceanogr..

[38] Widdig A, Schlichter D (2001). Phytoplankton: a significant trophic source for soft corals?. Helgol. Mar. Res..

[39] Colaço A, Giacomello E, Porteiro F, Menezes GM (2013). Trophodynamic studies on the Condor seamount (Azores, Portugal, North Atlantic). Deep. Res. Part II Top. Stud. Oceanogr..

[40] Addamo AM (2016). Merging scleractinian genera: the overwhelming genetic similarity between solitary Desmophyllum and colonial Lophelia. BMC Evol. Biol..

[41] Mueller CE, Larsson AI, Veuger B, Middelburg JJ, van Oevelen D (2014). Opportunistic feeding on various organic food sources by the cold-water coral *Lophelia pertusa*. Biogeosciences.

[42] Roushdy H, Hansen V (1961). Filtration of phytoplankton by the octocoral Alcyonium digitatum. Nature.

[43] Sorokin Y (1991). Biomass, metabolic rates and feeding of some common reef zoantharians and octocorals. Aust. J. Mar. Freshw. Resour..

[44] Seemann J (2013). The use of 13C and 15N isotope labeling techniques to assess heterotrophy of corals. J. Exp. Mar. Biol. Ecol..

[45] Orejas C (2016). The effect of flow speed and food size on the capture efficiency and feeding behaviour of the cold-water coral Lophelia pertusa. J. Exp. Mar. Biol. Ecol..

[46] Carmo V (2013). Variability of zooplankton communities at Condor seamount and surrounding areas, Azores (NE Atlantic). Deep. Sea Res. Part II Top. Stud. Oceanogr..

[47] Gori A, Grover R, Orejas C, Sikorski S, Ferrier-Pagès C (2014). Uptake of dissolved free amino acids by four cold-water coral species from the Mediterranean Sea. Deep. Sea Res. Part II Top. Stud. Oceanogr..

[48] Sweetman, A. K. *et al.* Major impacts of climate change on deep-sea benthic ecosystems. *Elementa**Science of the Anthropocene *vol. 5 (2017).

[49] Migné A, Davoult D (2002). Experimental nutrition in the soft coral Alcyonium digitatum (Cnidaria: Octocorallia): Removal rate of phytoplankton and zooplankton. Cah. Biol. Mar..

[50] Sebens KP, Koehl MAR (1984). Predation on zooplankton by the benthic anthozoans Alcyonium siderium (Alcyonacea) and Metridium senile (Actiniaria) in the New England subtidal. Mar. Biol..

[51] Gili J-M, Coma R, Orejas C, López-González P, Zabala M (2001). Are Antarctic suspension-feeding communities different from those elsewhere in the world?. Polar Biol..

[52] Rossi S (2006). Temporal variation in protein, carbohydrate, and lipid concentrations in Paramuricea clavata (Anthozoa, Octocorallia): evidence for summer-autumn feeding constraints. Mar. Biol..

[53] Coma R, Ribes M, Gili J-M, Zabala M (2000). Seasonality in coastal benthic ecosystems. Trends Ecol. Evol..

[54] Bythell JC, Wild C (2011). Biology and ecology of coral mucus release. J. Exp. Mar. Biol. Ecol..

[55] Brooke S, Holmes M, Young C (2009). Sediment tolerance of two different morphotypes of the deep-sea coral Lophelia pertusa from the Gulf of Mexico. Mar. Ecol. Prog. Ser..

[56] Larsson AI, van Oevelen D, Purser A, Thomsen L (2013). Tolerance to long-term exposure of suspended benthic sediments and drill cuttings in the cold-water coral Lophelia pertusa. Mar. Pollut. Bull..

[57] Ragnarsson, S. Á. *et al.* The impact of anthropogenic activity on cold-water corals. in *Marine Animal Forests: The Ecology of Benthic Biodiversity Hotspots* 989–1023 (Springer International Publishing, 2017). 10.1007/978-3-319-21012-4_27.

[58] Rix L (2016). Coral mucus fuels the sponge loop in warm- and cold-water coral reef ecosystems. Sci. Rep..

[59] Lampert W (1978). Release of dissolved organic carbon by grazing zooplankton. Limnol. Oceanogr..

[60] Moller EF (2004). Sloppy feeding in marine copepods: prey-size-dependent production of dissolved organic carbon. J. Plankton Res..

[61] Burton T, Killen SS, Armstrong JD, Metcalfe NB (2011). What causes intraspecific variation in resting metabolic rate and what are its ecological consequences?. Proc. R. Soc. B Biol. Sci..

[62] Burgess SC (2017). Metabolic scaling in modular animals. Invertebr. Biol..

[63] Maier SR (2019). Survival under conditions of variable food availability: Resource utilization and storage in the cold-water coral *Lophelia pertusa*. Limnol. Oceanogr..

[64] Okie JG (2015). Niche and metabolic principles explain patterns of diversity and distribution: theory and a case study with soil bacterial communities. Proc. R. Soc. B Biol. Sci..

[65] van Oevelen D (2009). The cold-water coral community as hotspot of carbon cycling on continental margins: a food-web analysis from Rockall Bank (northeast Atlantic). Limnol. Oceanogr..

[66] Cathalot C (2015). Cold-water coral reefs and adjacent sponge grounds: hotspots of benthic respiration and organic carbon cycling in the deep sea. Front. Mar. Sci..

[67] Coppari M, Zanella C, Rossi S (2019). The importance of coastal gorgonians in the blue carbon budget. Sci. Rep..

[68] Moller EF, Nielsen TG (2001). Production of bacterial substrate by marine copepods: effect of phytoplankton biomass and cell size. J. Plankton Res..

[69] Titelman J, Riemann L, Holmfeldt K, Nilsen T (2008). Copepod feeding stimulates bacterioplankton activities in a low phosphorus system. Aquat. Biol..

[70] Violle C, Jiang L (2009). Towards a trait-based quantification of species niche. J. Plant Ecol..

[71] Yesson C (2012). Global habitat suitability of cold-water octocorals. J. Biogeogr..

[72] Kearney M, Simpson SJ, Raubenheimer D, Helmuth B (2010). Modelling the ecological niche from functional traits. Philos. Trans. R. Soc. B Biol. Sci..

[73] Violle C (2007). Let the concept of trait be functional!. Oikos.

[74] Evans TG, Diamond SE, Kelly MW (2015). Mechanistic species distribution modelling as a link between physiology and conservation. Conservation Physiology.

[75] Johnson, J. Y. Description of a new species of flexible coral belonging to the genus Juncella, obtained at Madeira. *Proc. Zool. Soc. London* 505–506 (1863).

[76] Weinberg, S. & Grasshoff, M. *Gorgonias. El Mar Mediterraneo. Fauna, Flora, Ecologia. II/1. Guia Sistematica y de Identificacion.* (Ediciones Omega, 2003).

[77] Carpine, C. & Grasshoff, M. Les gorgonaires de la Méditerranée. *Bull. l’Institut Océanographique* 1–140 (1975).

[78] Brito, A. & Ocaña, O. *Corales de las Islas Canarias*. (2004).

[79] Cau A (2015). Deepwater corals biodiversity along roche du large ecosystems with different habitat complexity along the south Sardinia continental margin (CW Mediterranean Sea). Mar. Biol..

[80] Tempera F, Harris PT, Baker EK (2012). Mapping the Condor seamount seafloor environment and associated biological assemblages (Azores, NE Atlantic). Seafloor geomorphology as benthic habitat: geohab atlas of seafloor geomorphic features and benthic habitats.

[81] Santos M (2013). Phytoplankton variability and oceanographic conditions at Condor seamount, Azores (NE Atlantic). Deep. Sea Res. Part II Top. Stud. Oceanogr..

[82] Sorokin YI (1973). On the feeding of some scleractinian corals with bacteria and dissolved organic matter. Limnol. Oceanogr..

[83] Maier SR (2019). Survival under conditions of variable food availability: Resource utilization and storage in the cold-water coral *Lophelia pertusa*. Limnol. Oceanogr..

[84] Zuur AF, Ieno EN, Elphick CS (2010). A protocol for data exploration to avoid common statistical problems. Methods Ecol. Evol..

[85] Zuur AF, Ieno EN, Walker N, Saveliev AA, Smith GM (2009). Mixed Effects Models and Extensions in Ecology with R.

[86] Bates D, Mächler M, Bolker B, Walker S (2015). Fitting linear mixed-effects models using lme4. J. Stat. Softw..

[87] Pinheiro, J., Bates, D., DebRoy, S., Sarkar, D. & R Core Team. nlme: linear and Nonlinear mixed effects models. R package version 3.1–140. (2019).

