# Supplementary material for: Contrasting metabolic strategies of two co-occurring deep-sea octocorals
Source: Sci Rep. 2021 May 20;11:10633. doi: 10.1038/s41598-021-90134-5 (PMC8138010; doi:10.1038/s41598-021-90134-5)
Supplement: Supplementary file 1 — Supplementary Information. [file 41598_2021_90134_MOESM1_ESM.pdf]

Contrasting metabolic strategies of two co-occurring deep-sea octocorals

Rakka M., Maier S. R., Van Oevelen D., Godinho A., Bilan M., Orejas C., Carreiro-Silva M.

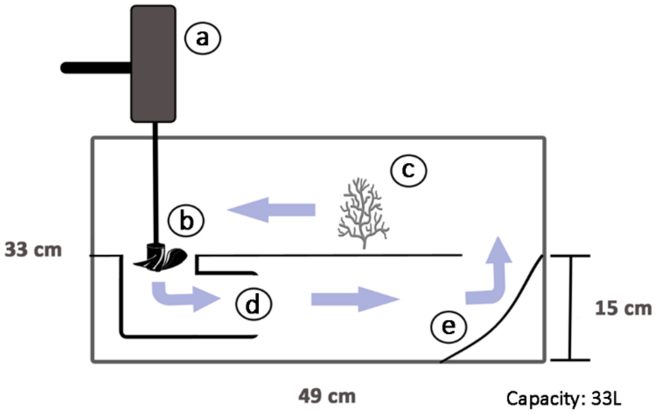

Figure S1: Experimental flumes used to determine the ability of the octocoral species *Dentomuricea* aff. *meteor* and *Viminella flagellum* to exploit different food substrates. Flume volume 33L. (a) Heavy duty motor; (b) 8 cm marine propeller; (c) coral fragment; (d) 9cm tube to direct flow; (e) platform used to facilitate flow direction.

Table S1: Coral colonies used in the feeding experiments, sampling locations and assignment of coral fragments to treatments and experimental aquaria

Species	Colony	Latitude	Longitude	Sampling depth (m)	Fragment	Treatment	Aquaria
D. aff. *meteor*	AC14	38.5406	-29.0541	190-230	AC14A	FAST	8
					AC14B	PHYTO	5
					AC14C	ZOO	7
					AC14D	ZOO	5
					AC14F	PHYTO	6
					AC14G	FAST	8
					AC14H	DOM	5
					AC14J	FAST	8
					AC14K	DOM	7
					AC14M	PHYTO	7
					AC14O	DOM	6
					AC14P	ZOO	6
	AC1	38.5770	-29.0180	230-240	AC1A	DOM	6
					AC1B	ZOO	5
					AC1D	PHYTO	5
					AC1E	PHYTO	6
					AC1F	DOM	5
					AC1H	FAST	8
					AC1I	ZOO	7
					AC1J	DOM	7
					AC1K	FAST	8
					AC1L	ZOO	6
					AC1O	FAST	8
					AC1P	PHYTO	7
	AC26	38.5406	-29.0541	190-230	AC26A	PHYTO	5

					AC26AB	ZOO	5
					AC26AH	DOM	7
					AC26AJ	DOM	6
					AC26AL	FAST	8
					AC26AM	ZOO	6
					AC26D	ZOO	7
					AC26K	PHYTO	6
					AC26L	FAST	8
					AC26P	PHYTO	7
					AC26Y	FAST	8
					AC26Z	DOM	5
	AC7A	38.5770	-29.0180	230-240	AC7A	FAST	8
					AC7AD	ZOO	7
					AC7E	FAST	8
					AC7F	PHYTO	5
					AC7G	PHYTO	7
					AC7I	DOM	5
					AC7L	ZOO	5
					AC7P	FAST	8
					AC7T	DOM	6
					AC7U	PHYTO	6
					AC7W	ZOO	6
					AC7X	DOM	7
	AC8A	38.5770	-29.0180	230-240	AC8A	ZOO	5
					AC8B	DOM	7
					AC8C	FAST	8
					AC8D	PHYTO	6
					AC8F	DOM	5
					AC8K	FAST	8
					AC8L	ZOO	6
					AC8M	PHYTO	7

					AC8N	DOM	6
					AC8O	ZOO	7
					AC8P	FAST	8
					AC8R	PHYTO	5
V. flagellum	AC10	38.5396	-29.0399	190-210	AC10C	FAST	6
					AC10D	PHYTO	7
					AC10L	ZOO	5
					AC10M	DOM	8
	AC11	38.5396	-29.0399	190-210	AC11A	DOM	5
					AC11B	FAST	6
					AC11C	PHYTO	7
					AC11G	ZOO	8
	AC12	38.5396	-29.0399	190-210	AC12A	PHYTO	7
					AC12C	FAST	5
					AC12E	DOM	8
					AC12F	ZOO	6
	AC13	38.5770	-29.0180	230-240	AC13A	FAST	8
					AC13B	PHYTO	6
					AC13C	ZOO	5
					AC13D	DOM	7
	AC15	38.5770	-29.0180	230-240	AC15A	PHYTO	6
					AC15B	DOM	7
					AC15C	ZOO	8
					AC15D	FAST	5
	AC16	38.5396	-29.0399	190-210	AC16A	DOM	8
					AC16D	ZOO	6
					AC16E	PHYTO	5
					AC16I	FAST	7
	AC17	38.5396	-29.0399	190-210	AC17A	PHYTO	8
					AC17D	ZOO	5
					AC17F	FAST	6

					AC17G	DOM	7
	AC27	37.6012	-25.8824	250-255	AC27A	DOM	5
					AC27B	PHYTO	8
					AC27D	ZOO	6
					AC27H	FAST	7
	AC29	37.6012	-25.8824	250-255	AC29A	PHYTO	5
					AC29D	FAST	7
					AC29F	ZOO	8
					AC29H	DOM	6
	AC30	37.6012	-25.8824	250-255	AC30A	PHYTO	5
					AC30B	DOM	6
					AC30C	FAST	8
					AC30D	ZOO	7
	AC9	38.5396	-29.0399	190-210	AC9A	DOM	6
					AC9B	ZOO	7
					AC9C	PHYTO	8
					AC9D	FAST	5
	AV9	38.5770	-29.0180	230-240	AV9A	PHYTO	6
					AV9B	ZOO	7
					AV9C	DOM	5
					AV9D	FAST	8

Table S2: Characteristics of coral fragments of the octocoral species *Dentomuricea* aff. *meteor* and *Viminella flagellum* used in the different experimental treatments.

	D.* aff. *meteor				***V.flagellum***			
	**FAST**	**PHYTO**	**DOM**	**ZOO**	**FAST**	**PHYTO**	**DOM**	**ZOO**
**Dry weight (g)**	0.28 ± 0.15	0.30 ± 0.16	0.29 ± 0.17	0.35 ± 0.17	1.58 ± 0.87	1.74 ± 1.14	1.44 ± 1.03	1.63 ± 0.93
**Skeletal weight (g)**	0.04 ± 0.02	0.04 ± 0.02	0.04 ± 0.02	0.05 ± 0.03	1.24 ± 0.79	1.41 ± 1.05	1.0 ± 0.87	1.31 ± 0.84
**Org. C content (%)**	7.09 ± 2.18	6.96 ± 1.96	7.03 ± 1.7	7.29 ± 1.25	7.36 ± 1.6	8.00 ± 1.7	8.22 ± 1.11	8.41 ± 1.40

Table S3: Summary of the model building procedure of Linear Mixed Effects models (LME) and Generalized Least-Square models (GLS) to investigate the effect of different food substrates (food treatments) on metabolic processes of the octocoral species *Dentomuricea* aff. *meteor* and *Viminella flagellum*. Model building was done by assessing the Akaike Information Criteria (AIC) for every model and the summaries of Likelihood Ratio Tests, including the L-ratio value, degrees of freedom (df) and p-value.

**Species**			***D.meteor***				***V.flagellum***			
**Variable group**	**Dependent variable**	**Independent factor**	**AIC**	**df**	**L ratio**	**p value**	**AIC**	**df**	**L ratio**	**p value**
**Tissue**	**Dry weight**	null	-68.98	3.00			106.62	3.00		
		Treatment	-66.10	6.00	3.12	0.37	110.39	6.00	2.23	0.53
	**Tissue weight**	null	-284.64	3.00			90.33	3.00		
		Treatment	-281.35	6.00	2.70	0.44	92.59	6.00	3.74	0.29
	**Skeletal weight**	null	-85.28	3.00			-33.21	3.00		
		Treatment	-82.73	6.00	3.44	0.33	-27.31	6.00	0.11	0.99
	**Organic carbon content**	null	192.35	3.00			167.74	3.00		
		Treatment	197.42	6.00	0.93	0.82	166.72	6.00	7.01	0.07
	**Nitrogen content**	null	31.35	3.00			56.90	3.00		
		Treatment	37.15	6.00	0.21	0.98	52.47	6.00	10.42	0.02
	**C/N**	null	33.84	3.00			-37.71	5.00		
		Treatment	23.90	6.00	15.94	0.00	-40.17	8.00	8.46	0.04
	**Tracer C incorporation**	null	236.94	5.00			111.01	4.00		
		Treatment	175.13	7.00	65.80	<0.0001	89.50	6.00	25.50	<0.001
	**Tracer N incorporation**	null	84.09	5.00			18.78	4.00		
		Treatment	16.52	7.00	71.56	<0.0001	1.89	6.00	20.80	<0.001

**Respiration**	**Oxygen consumption**	null	43.12	3.00			27.82	3.00		
		Treatment	36.80	6.00	12.20	0.01	13.49	6.00	20.33	0.00
	**DIC Bulk respiration**	null					-10.16	2.00		
		Treatment					-6.93	4.00	0.76	0.68
	**Tracer C respiration**	null	-33.83	3.00			-74.46	4.00		
		Treatment	-37.76	4.00	5.93	0.01	-80.16	6.00	9.70	0.01
**POC/PON Release**	**POC bulk release**	null	-30.56	5.00			-56.60	2.00		
		Treatment	-39.21	8.00	14.64	0.00	-55.22	4.00	2.61	0.27
	**Tracer C release**	null	-82.57	4.00			-156.50	4.00		
		Treatment	-86.93	6.00	8.36	0.02	-165.50	6.00	13.00	0.00
	**PON bulk Release**	null	-85.10	2.00			-110.05	4.00		
		Treatment	-85.20	5.00	6.13	0.11	-116.90	6.00	10.90	0.00
	**Tracer N excretion**	null	-128.76	4.00			-251.40	4.00		
		Treatment	-139.80	6.00	15.03	0.00	-255.40	6.00	7.94	0.02
